# Supporting Staff To Address the Physical Health of Consumers in Mental Healthcare Settings: A Modified Nominal Group Technique

**DOI:** 10.1007/s10597-025-01589-z

**Published:** 2026-01-22

**Authors:** Di Hu, Victoria Stewart, Amanda J Wheeler, Geoffrey Lau, Mary Lou Chatterton, Donni Johnston, Paul G Klotz, Nicole Korman, Oscar Lederman, Sam Manger, Irene McCarthy, Grace McKeon, Sally Plever, Amanda L Rebar, Russell Roberts, Simon Rosenbaum, Puspa Sherlock, Jennie Somerville, Shuichi Suetani, Scott Teasdale, Philip Ward, Andrew Watkins, Justin Chapman

**Affiliations:** 1 Centre for Mental Health, Griffith University, Brisbane, QLD Australia; 2 School of Pharmacy and Medical Sciences, Griffith University, Brisbane, QLD Australia; 3 Addiction and Mental Health Services, Metro South Health, Brisbane, QLD Australia; 4 Public Health and Preventive Medicine, Monash University, Melbourne, VIC Australia; 5 Faculty of Medicine, The University of Queensland, Brisbane, QLD Australia; 6 Independent Researcher, Bundaberg, QLD Australia; 7 Physical Health and Mental Health Stream, Queensland Centre for Mental Health Research, Wacol, Australia; 8 Human Performance Research Centre, School of Sport, Exercise and Rehabilitation, Faculty of Health, University of Technology Sydney (UTS), Moore Park, Sydney , NSW Australia; 9 Nutrition, Exercise & Social Equity (NExuS) Research Group, Discipline of Psychiatry and Mental Health, School of Clinical Medicine, University of New South Wales, Sydney, NSW Australia; 10 Lifestyle Medicine, James Cook University, Douglas, QLD Australia; 11 The Queensland Mental Health Clinical Collaborative, Metro North Hospital and Health Service, Brisbane, QLD Australia; 12 School of Public Health, The University of Queensland, Brisbane, QLD Australia; 13 Motivation of Health Behavior Lab; Health Promotion, Education, and Behavior; Arnold School of Public Health, University of South Carolina, Columbia, SC USA; 14 Motivation of Health Behaviour Lab; Appleton Institute; School of Health, Medical and Applied Sciences, Central Queensland University, Rockhampton, QLD Australia; 15 School of Business, Charles Sturt University, Bathurst, NSW Australia; 16 Independent Researcher, Brooklyn, TAS Australia; 17 Independent Researcher, Sydney, VIC Australia; 18 The University of Queensland, Brisbane, QLD Australia; 19 Schizophrenia Research Unit, South Western Sydney Local Health District and Ingham Institute of Applied Medical Research, Liverpool, NSW Australia; 20 Mindgardens Neuroscience Network, Randwick, NSW Australia

**Keywords:** Mental health, Physical healthcare, Nominal Group Technique (NGT), Theoretical Domains Framework (TDF), Quality Improvement (QI), Service improvement

## Abstract

Mental health consumers experience challenges in accessing regular physical healthcare. Staff working in mental health settings are well-positioned to address the issue; however, they often face significant barriers in delivering physical healthcare. The primary aim of this study was to identify important factors that support mental health staff to address the physical health of consumers; the secondary aim was to evaluate the utility of using a modified nominal group technique (NGT). Four main methodological modifications to standard NGT methods were made: (i) theoretical models (COM-B and Theoretical Domains Framework) were used to frame the NGT process, (ii) idea generation and ranking steps were conducted outside NGT sessions, (iii) both in-person and online groups were offered, and (iv) participants contributed to interpretation and dissemination of the findings. Individuals with expertise in the mental health sector participated in one of two NGT sessions, either in-person (n=13) or online (n=8). Considering Australian public mental health services, participants responded to the question: “What is needed for staff to optimally address physical health within their role?”. Additionally, participant experiences and acceptability of this modified NGT process were evaluated through follow-up interviews (n=6). A total of 55 concepts were generated within COM-B components: capability (n=18), opportunity (n=26), and motivation (n=11). Highest-ranking key concepts were awareness (capability), leadership support (opportunity), and beliefs (motivation). Qualitative data indicated that the modified NGT method was effective, particularly when using a framework to guide ideas generation; however, the additional time commitment may reduce feasibility.

**Supplementary Information:**

The online version contains supplementary material available at 10.1007/s10597-025-01589-z.

## Introduction

People experiencing mental illness, such as schizophrenia and bipolar disorder (herein described as mental health consumers) have a higher mortality rate compared with the general population (Walker et al., 2015). Their life expectancy is often 10 to 20 years less than the general population, largely due to physical health issues, in particular cardiovascular disease, respiratory illness, cancer, and metabolic diseases (Chesney et al., 2014; Firth et al., 2019; Lawrence et al., 2013). Mental health consumers are twice as likely to experience these chronic physical health issues when compared to the general population (Halstead et al., 2024). Some of these conditions are commonly associated with metabolic side-effects of mental health medications, and further compounded by socioeconomic and behavioural factors, including sedentary activity, smoking, poor nutritional intake, and social disadvantages (De Hert et al., 2011). While the provision of integrated physical healthcare interventions to mental health consumers has been found to significantly improve their health outcomes (Tuudah et al., 2022), this population continues to face challenges in accessing regular physical health checks and screenings compared to those without mental illness (Liu et al., 2017).

Fragmentation of health services and limited mental health workforce capability in addressing the physical health of consumers are systemic obstacles to improving health outcomes for mental health consumers (Duggan, 2015; Firth et al., 2019). The separation of physical and mental healthcare, or the “siloing” of healthcare provision, reduces consumers’ access to adequate health services, contributing to poor physical health outcomes (Happell et al., 2012). Diagnostic overshadowing occurs when physical symptoms are mistakenly attributed to a person’s mental health condition, which can result in overlooked or misdiagnosed health problems and contribute to health disparities (Jones et al., 2008). Moreover, resource restraints in mental health settings limit workforce capacity to address the physical healthcare needs of consumers (Clancy et al., 2019). These obstacles decrease the rates of monitoring and management of physical health issues for this population (Lawrence & Kisely, 2010). For example, people experiencing mental illness are less likely to receive surgical interventions (McBride et al., 2021), standard diabetes care (Prathiksha et al., 2024), and routine cancer screening (Murphy et al., 2021) when compared with the general population.

Staff working in mental health settings have access to mental health consumers and hence are well-positioned to address the physical health issues of consumers (Lamontagne-Godwin et al., 2018); however, they face significant barriers to delivering physical healthcare to this group (Watkins et al., 2020). For example, staff working in mental health settings often lack knowledge of physical health issues, and available physical healthcare resources (Bartlem et al., 2020; Happell et al., 2012; Tredget et al., 2023), leading to low confidence in delivering physical healthcare to consumers (Gunasekaran et al., 2022) and misperceptions about consumers being resistant to such services (Clancy et al., 2019). Moreover, the heavy workload of mental health staff and lack of clarity about the professional responsibility of physical healthcare within their roles limits their capacity to provide physical healthcare (Watkins et al., 2023). Because of these factors, organisational culture in mental health settings is often not conducive to the provision of physical healthcare (Ehrlich et al., 2015). While some of these barriers may have limited modifiability (e.g., mental health service funding), others may be influenced by using Quality Improvement (QI) methods to alter staff practices in the provision of physical healthcare for this population.

QI is an integrated and interdisciplinary approach that aims to enhance consumer outcomes, system performance, and professional development, emphasising iterative change, learning, and adaptation (Backhouse & Ogunlayi, 2020). It is a systematic process that targets the gap between evidence and practice by introducing innovations designed to improve system and workforce capacity (Forman-Hoffman et al., 2017). QI interventions often aim to change staff behaviour in the given area of practice to improve the quality of healthcare, and can benefit from being designed using behavioural theory such as the Behaviour Change Wheel and COM-B model (Mather et al., 2022). The COM-B is a model of behaviour in which **C**apability (physical and psychological), **O**pportunity (physical and social), and **M**otivation (automatic and reflective) interact to produce **B**ehaviour (Michie et al., 2011). The COM-B model extends from the Theoretical Domains Framework (TDF) (Michie et al., 2005), and was developed to understand behaviour change in implementation. The TDF outlines 14 domains related to the success or failure of any health service implementation project, including knowledge, skills, professional identity, beliefs, environmental resources, etc. (Michie et al., 2005). The COM-B model and TDF can be used to better understand what factors could be targeted by QI interventions to support staff in addressing the physical health of consumers.

The Nominal Group Technique (NGT) method has demonstrated effectiveness in bridging theories with practical insights of experts from mental and physical healthcare backgrounds (Smith et al., 2024). The NGT is a method of generating consensus with diverse stakeholders, increasing the likelihood of identifying the key factors related to a particular question (Olsen, 2019). It is a systematic and well-established method designed to generate (Søndergaard et al., 2018) and prioritise solutions to a specific question, identifying those that will be more likely to lead to better outcomes. The ideal participants for NGT should be those who have relevant knowledge and experience of the topic, including individuals with a diversity of characteristics to obtain a variety of perspectives (Cooper et al., 2020; Sanders, 2008). A traditional NGT process includes four key steps designed to give all participants an equal opportunity to contribute (Pokorny et al., 1988). First, participants consider the NGT question and prepare their ideas independently (silent generation of ideas), then take turns presenting them to the group (round robin). Once no new ideas have been generated, they are discussed and clarified amongst the group (clarification), and the final list is ranked anonymously (ranking). The NGT has been widely used for consensus building in diverse topic areas, such as preferences for end-of-life care (Dening et al., 2013) and nursing competency improvement (Foth et al., 2016), and may be useful for better understanding what factors may be targeted using QI interventions to support physical healthcare in mental health settings.

This NGT study identifies factors needed for mental health staff to provide physical healthcare using behaviour change theory to inform design of further physical healthcare QI interventions in mental health settings. The primary aim of this study was to generate consensus on the factors needed to support staff to address the physical health of consumers in mental health settings. A secondary aim was to evaluate participant experiences and acceptability of this modified NGT method.

## Method

### Participant Recruitment

Individuals with expertise in improving the physical health outcomes for mental health consumers were invited to participate via email on July 4th 2023, including mental health workers, academics, and those with a lived experience of mental illness. They were identified through the research team’s networks, the Equally Well initiative—a national program focused on improving the quality of life for mental health consumers, and authorship of relevant studies, such as *The Lancet Psychiatry Commission: A Blueprint for Protecting Physical Health in People with Mental Illness (*Firth et al., 2019). Participants were offered a $50 gift card or the option to donate $50 to a designated charitable organisation, and the option to be involved in the study as co-authors, provided they engaged in ongoing collaboration to meet authorship criteria. Those who provided written informed consent were given an overview of the NGT method, and a factsheet about the COM-B model and TDF, to assist them in considering different factors that would answer the NGT question:*‘What is needed for staff to optimally address physical health within their role?’*. Participants were also asked to answer demographic questions (professional background, mental health working experience, lived experience, and gender). Ethics approval was obtained from the University’s Human Research Ethics Committee.

## NGT Modifications

Traditional NGT methods have not used theoretical models to frame the idea generation, clarification, and ranking processes. However, incorporating the COM-B model and the TDF can strengthen the focus on behaviour change in service improvement, which aligns closely with the aims of this research. The COM-B model conceptualises capability, opportunity, and motivation as independent but related influences on behaviour; therefore, ranking ideas across these components is conceptually difficult, and it was important to modify the traditional NGT model to rank within each component. Modifications to the NGT method are described in Table 1.

**Table 1:** Overview of the nominal group technique (NGT) modifications

No.	Modification in this NGT	Traditional NGT	Reason for modification
1	COM-B* model and TDF^#^ used to frame the NGT process for idea generation and clarification steps, and subsequent interpretation of participant contributions and ranking within each COM-B component.	Traditional NGT procedures have not used theoretical models to prompt participants or separate the ideas generated for ranking.	Human behaviour is dependent on all three Capability, Opportunity and Motivation components. Ranking ideas within each component was considered more sensible rather than trying to rank across these independent domains.
2	The *silent generation of ideas*, *clarification*, and *ranking* steps were conducted outside the NGT meetings.	Silent generation of ideas to respond to the nominal question, and ranking conducted during the meeting.	Due to the time constraints, participants were asked to complete the silent generation of ideas step prior to the meeting. Clarification of ideas required ideas synthesis using the COM-B model and TDF, which could not be completed during the session.
3	Two different formats for conducting the NGT sessions were used: one online and one in-person.	Traditional NGT processes use highly structured face-to-face group interactions. Few studies have used online NGT sessions.	To improve accessibility and ensure participants from diverse geographical regions in Australia were able to participate.
4	Participants were engaged as team members to assist with analysis and interpretation of their contributions after the NGT sessions concluded.	The traditional NGT group is finished after participants rank ideas.	Clarification and ranking steps required ongoing interpretation and input from participants outside NGT sessions to allow sufficient consideration using the COM-B and TDF models.

*COM-B: Capability, Opportunity, and Motivation interact to produce Behaviour

^#^TDF: Theoretical Domain Framework

## NGT Procedure

Two NGT sessions were conducted in 2023: a 2.5-hour in-person meeting in July, and a 2-hour online meeting in September, facilitated by the lead author and another researcher skilled in facilitation processes. Both meetings used the same process of idea generation, round robin, and clarification to generate ideas with participants, and were audio-recorded and transcribed.

The lead author reviewed the transcripts to ensure all ‘ideas’ (raw data) discussed during the NGT meetings were captured. Furthermore, the lead author and two researchers collaboratively discussed and synthesised these ideas, which were then categorised into the COM-B and TDF constructs. Participants were asked to provide feedback on the interpretation of ideas and accuracy of categorisation via an online survey with 82 open-ended questions, using a research survey tool—LimeSurvey. These three researchers discussed any amendments, and revised categorisations based on participant feedback.

The researchers then synthesised the ideas into a final set of concepts in each of the three COM-B components. Once finalised, participants were asked to rank the top five concepts in each component in terms of importance; the top-ranked concepts from each person were allocated a score for analysis (range = 5 to 1; 5 = highest ranked concept) see Table 3. This ranking survey was designed using a research survey tool—Qualtrics. Participants were asked to select their five most important concepts for each component and rank them in order of importance for supporting staff in providing physical healthcare within mental health settings. Finally, summary statistics were generated for demographic data, and COM-B and TDF categories related to all ideas generated in the NGT sessions.

## Evaluation of Participant Experiences

**Fig. 1:**
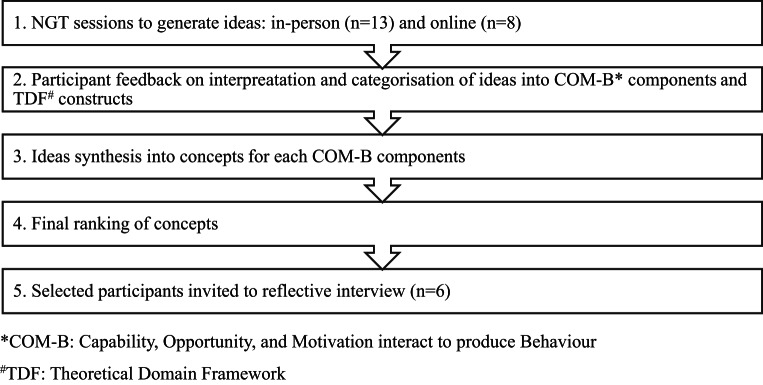
Nominal group technique (NGT) study procedure

A sub-sample of participants from the in-person (*n* = 3) and online (*n* = 3) NGT meetings were invited to participate in a short semi-structured interview (≈ 30 min) to gain their perspectives on the modified NGT process. Participants were selected based on their contribution to the overall study, including the level of involvement and the amount of interaction with data and feedback they provided. Interviews were held online through Microsoft Teams by the lead author and recorded for data analysis purposes. During the interview, participants were asked to describe their experiences with the study and report on steps and processes of the NGT to evaluate the acceptability of the modified NGT method. Qualitative data from feedback interviews were analysed descriptively, focused on the research questions e.g., the NGT process, utility and understanding of the theoretical models underpinning the NGT, and processes that supported ongoing involvement. The lead author and one researcher independently read the transcripts and met to generate a coding framework. Data was organised using NVivo 14. The overall NGT study process is listed in Fig. 1.

## Results

Overall, 21 participants consented and attended an NGT session (*n* = 13 in-person; *n* = 8 online); however, one participant withdrew after completing the in-person NGT session due to conflicting commitments. Most participants were female (*n* = 12, 57%), and most had over 10 years experience working in the mental health sector (*n* = 18, 90%). Participants worked in a clinical setting (*n* = 9), academic (*n* = 4), in both settings (*n* = 4), or as a lived experience consultant or mentor (*n* = 3); six participants reported a lived experience of mental illness, and one as a carer.

### Factor to Support Staff in Addressing Physical Healthcare for Mental Health Consumers

Analysing raw data from the NGT transcripts identified 82 unique ideas, which were categorised into COM-B components and TDF domains. Participants provided feedback on the interpretation and categorisation of each idea: a total of 308 comments were provided, 231 (75%) of which were in agreement, 45 (15%) suggested additional information to improve the clarification of ideas, and 32 (11%) suggested re-categorising the idea into different COM-B categories. The final categorisations are presented in Table 2: the highest number of ideas was in the Opportunity COM-B component (*n* = 80, 57%), and the most frequent TDF domains were *skills* in Capability, *environmental context and resources* in Opportunity, and *beliefs about capability* in Motivation. Some ideas were categorised into multiple COM-B components and TDF domains; for example, TDF domains *skills* and *knowledge* in providing physical healthcare were identified in relation to communication skills and knowledge of person-centred language:

*[“I certainly think it needs to change the language with how we’re communicating around physical health and the connection to mental health … we can actually have these conversations using much more inclusive and person-centred language.”]*.

Some ideas included the content of two COM-B components; for example, an idea included *knowledge* in Capability and *Social professional role identity* in Motivation:

*[“I think having that systemic knowledge of this and confidence in being able to deliver lifestyle recommendations before coming into these positions.”]*.

**Table 2:** The frequency of ideas related to each TDF domain in COM-B components

COM-B Components	TDF Domains	Number of ideas
Capability	Skills	22
	Knowledge	18
	Behavioural regulation	2
	Memory, attention, and decision	1
	Generic^#^	2
	***Capability total***	***45 (32%)***
Opportunity	Environmental context and resources	74
	Social influence	6
	***Opportunity total***	***80 (57%)***
Motivation	Beliefs about capability	5
	Social professional	4
	Beliefs about consequences	2
	Intentions	2
	Reinforcement	2
	Emotion	1
	***Motivation total***	***16 (11%)***
Total		**141 (100%)**

Some of the 82 unique ideas overlap with COM-B components and TDF domains; therefore, the total number of ideas relevant to each domain exceeded 82

^#^Generic idea demonstrates general information, like building staff capacity

The 82 unique ideas on supporting staff to provide physical healthcare to mental health consumers were synthesised into 55 concepts for ranking, and included 18 in Capability, 26 in Opportunity, and 11 in Motivation. Of the 21 participants, 11 completed the survey with 82 open-ended questions to obtain detailed feedback on the interpretation and categorisation of ideas into COM-B components and TDF constructs. Moreover, the ranking survey was completed by 19 of the 21 NGT participants. The number of votes and total ranking score for the top five concepts are presented in Table 3, with a list of all rankings provided in the supplementary material (Supplementary 1).

**Table 3:** The number of votes and total score for the top 5 ranked concepts

COM-B components	Rank	Concepts	Total score	Voting counts
Capability	First	Awareness of their responsibility in providing physical healthcare in mental health settings.	39	10
	Second	Ability to listen and communicate about physical health with consumers in mental health settings.	27	9
	Third	Knowledge of available physical healthcare referral pathways.	27	8
	Fourth	Skills in identifying consumers at risk, and responding with physical health prevention services.	23	9
	Fifth	Skills in providing person-centred physical healthcare in mental health settings.	23	7
Opportunity	First	Leadership support to prioritise, guide and support staff to provide physical healthcare.	42	10
	Second	Organisational culture in mental health settings that prioritises physical health.	27	9
	Third	Funding that supports staff to deliver evidence-based physical health interventions and quality improvement.	26	7
	Fourth	Organisational accountability to ensure physical healthcare can be provided within staff workload capacity.	24	7
	Fifth	Staff positions which are dedicated to addressing physical health in mental health settings.	23	7
Motivation	First	Believing that physical healthcare can contribute to mental health recovery.	46	11
	Second	Physical healthcare has become habitual and integrated into daily practice.	38	12
	Third	Influence of role models or champions in physical healthcare.	38	12
	Fourth	Physical healthcare leadership identity in mental health settings.	33	10
	Fifth	Professional confidence in working effectively with consumers to facilitate behaviour change.	29	10

## Evaluation of Participant Experiences and Acceptability of the Modified NGT Experience

A total of six participants completed an interview, with an average duration of 15.8 min (range = 9.8 to 29 min). Four were health practitioners, and two were academics, with most having 10–20 years of experience in the mental health sector; three were male and three were female. Two also reported having a lived experience of mental illness. An analysis of their responses is provided below.

Participants reported that the overall NGT study was well-facilitated and the modifications were acceptable. *[It can be new or slightly different to what a lot of people are used to*,* but in my mind*,* it makes sense. (P1)]* Preparing for the NGT meeting was seen as important, particularly understanding the underpinning COM-B and TDF frameworks. However, challenges in completing the preparation activities during busy work schedules resulted in some participants not reading the pre-NGT documents or preparing their responses. *[So in terms of like just the feasibility and practicality of it*,* if you’re asking people to do homework before or after something*,* it probably won’t happen unfortunately. (P2)]* This was particularly evident during the idea generation session, with most unaware of the COM-B and TDF frameworks *[I wasn’t as aware of COM-B (P4) … because we’re doing a lot of brainstorming*,* it was kind of more like just sort of scatter-gunning ideas. (P4)]* Therefore, recommendations to use the COM-B and TDF frameworks during the data analysis were made by participants, reducing the need to categorise ideas during the meetings. *[In a focus group like what we had*,* it’s not forcefully put into those frameworks*,* rather that’s what the data analysis… could kind of categorise it into those areas.(P2)]* While the process of using the frameworks during the NGT was challenging, most participants felt there were benefits in using a theory-driven approach.

Participants identified that attending the NGT meetings with people from a range of locations was important, as it captured the different perspectives that motivated others to learn and generate more new ideas.

*[I think that was great to get a range of different people from different parts of Australia*,* different perspectives*,* different experiences in the one room. I want to sort of use each other’s experiences and energy and work off that and the follow up stuff from that. (P1)*

*It was interesting because my background is in motivation*,* science and theory*,* so I tend to think kind of in a different direction than a lot of the other experts because they were more on the ground and thinking of implementation where I was going theory up. (P6)]*

However, participants also noted that being unfamiliar with other participants in the meeting made it difficult to share openly. Those who attended online NGT meetings reported feeling more comfortable.

*[Comfortable using the online format… you don’t have to wait necessarily you can put things in the chat and that was useful. (P6)]*

Facilitation of NGT meetings was identified as important, enabling equal opportunity to voice new ideas, *[Facilitation of that workshop enabled the power to be shared … so many important statements that are captured. (P1)]* and group discussions enhanced their participation and contribution.

*[Group to decide and to come up with the ideas and rank them … that was really well done and made my participation. (P1)]*

Participants attending the in-person NGT meeting recommended increasing the duration to ensure all ideas were heard. [*The time was a little rushed… have enough time to understand all of the ideas participants generated during the meeting. (P5)]* On the other hand, those who attended the 2-hour online meeting found it long and difficult to fit into their busy work schedules. *[Online meeting … was like two hours … is a pretty long time to get a lot of academics together … people are very time poor. (P2)]* The importance of providing incentives to motivate participation was recognised as ongoing contribution to the NGT study was found to be time-consuming and required significant commitment.

*[The obvious incentive of being an author … that academics kind of have to go by*,* there needs to be that incentive. (P2)]*,

Participants reported that ranking the ideas and identifying the priorities were essential in the NGT study. However, they also faced challenges in the prioritisation process, particularly due to the number of items and overlapping content. This resulted in significant time investments *[I found quite tricky trying to order one to five … some of them were overlapping. (P4) I couldn’t differentiate which were the priority because I felt like they were all very important. (P2)]* Participants also noted that some people may have different understandings of the items based on their backgrounds, which impacted the prioritisation process.

*[The main challenges is just understanding the different people have different language than the way they use things … consensus is going to be hard to achieve in some of those areas. (P3)]*

Participants demonstrated that ongoing contribution to the NGT study included many unexpected time-consuming steps that needed commitment following the group session. Whilst some steps were at times overwhelming, they felt they were reasonable to ensure the accuracy of the categorisation process. Additionally, clear instructions and quality information to guide their participation were recommended. *[Transcript information was a bit confusing … a couple of the comments were quite hard to understand (P4)]* Moreover, they identified some technical issues with the online surveys timing-out and resulting in the work they contributed not being saved. Most participants didn’t have time to complete the work again; therefore, some crucial feedback was missed.

*[I’d spent a bit of time on it and then it didn’t save … I probably only ended up adding about 30% or 40% of what I did in because I couldn’t remember (P3)]*

## Discussion

The most important factors needed to support mental health staff to address the physical health needs of mental health consumers were identified and prioritised in this NGT study from the perspective of experts working in the mental health field. The COM-B model and TDF were employed to frame the NGT process and provide a complete picture of behaviour change influences by focusing on capability, opportunity, and motivation, and the TDF was used to categorise domains related to service improvement and implementation behaviour. Most ideas suggested by participants related to Opportunity (56% of ideas and 47% of concepts), and the least related to motivation (11% of ideas and 20% of concepts).

Concepts in Capability highlighted the awareness, skills, and knowledge of staff to deliver physical healthcare in mental health settings. The need for staff to be aware of their responsibilities in delivering physical healthcare in mental health settings was prioritised as the most important factor in Capability, highlighting its importance in successful service implementation (Nzuva & Kimanzi, 2022). Whilst physical healthcare has been identified within the scope of practice of many mental health practitioners (Galletly et al., 2016), barriers such as lack of professional training, limited resources, and lack of clarification of staff responsibilities have been identified as barriers (Butler et al., 2020). Effective communication was also identified as a priority, using accessible language to communicate about physical healthcare with consumers effectively enhances therapeutic relationships (Bright & Reeves, 2022). Additionally, person-centred physical healthcare which considers consumers’ values, preferences, and needs, was prioritised. This approach has been found to improve engagement in change and the empowerment of consumers (Ekman et al., 2011; Van Dulmen, 2011).

Identifying consumers who are at risk of physical health issues and providing preventative physical healthcare, such as physical health checks and referrals to physical health programs or support services, were emphasised in the third- and fourth-ranked concepts in Capability. Previous research has demonstrated that preventative physical healthcare is an effective approach to improving the physical health of consumers with mental health conditions (Happell et al., 2012), with mental health staff serving as a link between mental and physical healthcare by referring appropriately (Hartley et al., 2020). This is crucial in reducing inequities in health outcomes for this population (Happell et al., 2012). According to the Behaviour Change Wheel, training is a significant behaviour change intervention function (Michie et al., 2011) and a popular approach used to enhance staff capacity for service improvement (Van Assen, 2021). However, training alone for the workforce may have limited effectiveness unless it is accompanied by ongoing support and leadership from management, which are important for transferring knowledge and skills into practice (Jabbie et al., 2024). It is, therefore, important that physical healthcare improvement programs should not only provide training but also incorporate ongoing support and management leadership, which target appropriate referral pathways to enhance health prevention interventions.

Concepts in Opportunity identified the essential role that services/organisations, and more broadly policy, play in prioritising and enabling staff to address the physical health of consumers. In line with the Behaviour Change Wheel, environmental restructuring creates conditions that allow for easier adoption and maintenance of behaviour (Michie et al., 2011). The top-ranked concepts highlighted opportunities such as leadership, organisational culture, financial support and designated positions to enable staff to deliver effective physical healthcare in mental health settings. Effective leadership and organisational culture empower staff behaviour change (Fitzgerald et al., 2013), and adequate funding provides staff with the time to learn about service innovations and document outcomes within workloads (Cowie et al., 2020). Identifying staff who have interests and enthusiasm in physical healthcare practices, and providing them with leadership development pathways could significantly improve individual capabilities, as well as enhance organisational culture and knowledge diffusion (Kjellström et al., 2020). Physical healthcare specialist positions were also identified, addressing gaps in physical health knowledge and attitudes for mental health staff as well as being a referral pathway to provide support (Fehily et al., 2023).

Concepts in Motivation focused on staff beliefs, habits, and confidence to deliver physical healthcare in mental health settings. Belief and behaviour change in service innovation is a complex process that requires individuals to understand and assess the benefits of changing their behaviour to deliver such innovations effectively (Alyafei & Easton-Carr, 2024). Staff need to understand the inter-relationship between physical and mental health, recognise the benefits of providing physical healthcare for mental health recovery, perceive providing this service as their responsibility, and prioritise physical healthcare amidst competing priorities in mental health settings. Beliefs are categorised as reflective motivation in the Behaviour Change Wheel, affected by intervention functions including education, persuasion, coercion, and incentivisation. Role modelling (Ee et al., 2020) and leadership (Fenwick et al., 2019) were ranked third and fourth, and are critical motivators that encourage staff behaviour change, enhance performance and positively influence colleagues (Alsadaan et al., 2023). In particular, a leadership concept identified during the NGT referred to having a physical health leadership identity. A strong sense of identity integrates and reflects core values and behaviours, enhancing performance and positively influencing colleagues (Alsadaan et al., 2023). This nuanced learning extends our understanding of how leadership can support staff behaviour change. Additionally, the NGT experts reported that physical healthcare needs to become habitual and integrated into daily practice (ranked second in Motivation). This requires supportive opportunities such as clear guidelines for staff responsibility, performance indicators, effective workforce management, and continuous service improvement (Monaghan & Cos, 2021). Low confidence can significantly impact the implementation of interventions (Hyland et al., 2003; Organ et al., 2010) and targeted training is needed to enhance staff knowledge and competence (Watkins et al., 2017).

This study employed a modified NGT process, including online and in-person sessions, silent generation and ranking outside the NGT meetings, and involving participants in the data analysis phase. The modified NGT process was deemed acceptable to participants and effective in achieving the study’s primary aim. Effective facilitation of the NGT groups ensured participants from diverse backgrounds and geographical locations were able to participate. Participants’ feedback indicated a preference for attending the online NGT meeting, noting that being unfamiliar with others in the in-person session made it harder to share openly. This aligns with previous findings that in-person NGT sessions may introduce power differentials and discourage contributions from those with less power (Khurshid et al., 2023). Ongoing contributions after the meetings supported the analysis and interpretation of results and led to collectively developed recommendations, particularly through the lengthy open-text survey used to collect feedback on concept interpretation and categorisation. However, these activities were time-consuming and sometimes affected by technical issues, which discouraged continued involvement. Offering participants the opportunity to collaborate as researchers and contribute as co-authors helped sustain their engagement throughout the study. While using theory to underpin the NGT was valued, this was a time-consuming process that may reduce feasibility. Therefore, participants suggested that it would be more practical for the research team to apply the frameworks during the data analysis phase instead.

### Strengths and Limitations

Strengths of this study include the utilisation of COM-B and TDF to frame the development of professional consensus, leading to theory-informed recommendations to practitioners and policymakers working in the mental health field. This study included a small sample of adults with experience in the Australian mental health sector. While this sample size is consistent with recommendations for NGT studies (Fox, 1989; Roth et al., 1995), findings may not be generalisable to international contexts or other service settings such as primary care. The NGT sessions were relatively short, and some concepts may have been missed because not all participants completed the idea generation step prior to the meeting. The two NGT groups were conducted in quite different formats (online and in-person), which may have introduced variability in how participants engaged with the consensus building process.

## Conclusion

This modified NGT study explored key factors that enable staff to provide physical healthcare in mental health settings, categorised according to the three components of the COM-B model: Capability, Opportunity, and Motivation. Opportunity had the highest number of identified concepts, while motivation had the fewest. The top priorities within the COM-B model highlighted the importance of leadership support (Opportunity), staff awareness of responsibility (Capability), and their beliefs (Motivation) in delivering physical healthcare in mental health settings. Feedback from participants identified strengths and challenges of the modified NGT study, which need to be considered in future studies. Using COM-B and TDF during the idea-generation step enhanced the quality of brainstorming; however, their application during data analysis required additional time commitment, impacting the feasibility of the modified NGT process. Overall, the findings from this modified NGT study can inform the evaluation of readiness for change for quality improvement in this area, and the design of future quality improvement interventions and evaluation of their impact.

## Supplementary Information

Below is the link to the electronic supplementary material.

Supplementary file 1 (DOCX 36.0 KB)

## Data Availability

The data generated in this study are available upon reasonable request.
