# Supplementary material for: Supporting Staff To Address the Physical Health of Consumers in Mental Healthcare Settings: A Modified Nominal Group Technique
Source: Community Ment Health J. 2026 Jan 22;62(5):998–1009. doi: 10.1007/s10597-025-01589-z (PMC13332962; doi:10.1007/s10597-025-01589-z)
Supplement: Supplementary file 1 — (DOCX 36.0 KB) [file 10597_2025_1589_MOESM1_ESM.docx]

**Supplementary 1. The number of votes and total score for all ranking concepts.**

| COM-B components | Rank | Concepts | Total score | Voting counts |
| --- | --- | --- | --- | --- |
| Capability | **1** | Awareness of their responsibility in providing physical healthcare in mental health settings. | 39 | 10 |
|  | **2** | Ability to listen and communicate about physical health with consumers in mental health settings. | 27 | 9 |
|  | **3** | Knowledge of available physical healthcare referral pathways. | 27 | 8 |
|  | **4** | Skills in identifying consumers at risk, and responding with physical health prevention services. | 23 | 9 |
|  | **5** | Skills in providing person-centred physical healthcare in mental health settings. | 23 | 7 |
|  | **6** | Knowledge of accessible physical health supports in the community. | 22 | 6 |
|  | **7** | Skills in prioritising elements of physical healthcare tailored to the needs of consumers with complex needs. | 22 | 10 |
|  | **8** | Knowledge of human rights and basic needs for physical healthcare in mental health settings. | 17 | 5 |
|  | **9** | Skills in holistic (whole-of-person) assessment and care in mental health settings. | 16 | 5 |
|  | **10** | Skills in creatively integrating physical healthcare within existing job responsibility. | 13 | 4 |
|  | **11** | Skills in addressing long-term physical health impacts (e.g., monitoring and treatment planning). | 12 | 5 |
|  | **12** | Staff capability (knowledge, skills, behavioural decisions) to live a healthy lifestyle. | 11 | 3 |
|  | **13** | Leadership skills in guiding team members in mental health settings to provide physical healthcare. | 9 | 4 |
|  | **14** | Skills in providing practical 'real-world' advice and education on healthy lifestyle behaviours. | 8 | 4 |
|  | **15** | Person-centred, inclusive, and common language used for communicating physical health in mental health settings. | 7 | 2 |
|  | **16** | Skills in recommending physical health activities based on consumers' preference. | 5 | 2 |
|  | **17** | Skills in collaborative care for physical and mental health. | 4 | 2 |
|  | **18** | Awareness of, and ability to challenge, personal biases/ stigma. | 0 | 0 |
| Opportunity | **1** | Leadership support to prioritise, guide and support staff to provide physical healthcare. | 42 | 10 |
|  | **2** | Organisational culture in mental health settings that prioritises physical health. | 27 | 9 |
|  | **3** | Funding that supports staff to deliver evidence-based physical health interventions and quality improvement. | 26 | 7 |
|  | **4** | Organisational accountability to ensure physical healthcare can be provided within staff workload capacity. | 24 | 7 |
|  | **5** | Staff positions which are dedicated to addressing physical health in mental health settings. | 23 | 7 |
|  | **6** | Involving lived experience workforce to increase the focus on holistic assessment and care, including physical health. | 15 | 6 |
|  | **7** | Compulsory performance indicators about physical health assessment and intervention. | 13 | 6 |
|  | **8** | Resource for linking and navigating physical healthcare services and supports in the community (digital system, or staff member). | 13 | 5 |
|  | **9** | Mandatory physical health training for mental health workforce. | 12 | 4 |
|  | **10** | Policies, guidelines and local operational procedures that support physical healthcare. | 10 | 3 |
|  | **11** | Undergraduate education on physical health for people with mental health concerns. | 10 | 4 |
|  | **12** | Accessible physical health equipment (e.g., blood pressure monitor, ECGs, spirometers). | 9 | 3 |
|  | **13** | Available professional training in physical health assessment and intervention. | 9 | 4 |
|  | **14** | Functional infrastructure for record keeping, data sharing, and evaluation. | 8 | 2 |
|  | **15** | Collaborative care models that support physical health in mental health settings. | 7 | 2 |
|  | **16** | Research data and policy facilitates physical healthcare in mental health settings. | 7 | 2 |
|  | **17** | Establish closer links between mental health services and community resources. | 6 | 2 |
|  | **18** | Equal access to community physical health resources and services in regional and remoted areas. | 5 | 2 |
|  | **19** | Organisational processes that reward provision of physical healthcare innovations. | 5 | 2 |
|  | **20** | Staff health and wellbeing programs. | 4 | 2 |
|  | **21** | A directory of accessible physical health supports and healthcare referral pathways. | 3 | 2 |
|  | **22** | Inter-professional learning opportunities for undergraduates and post-graduates. | 3 | 1 |
|  | **23** | Accessible database or collection of physical health information resources (e.g. handouts, manualised interventions). | 2 | 1 |
|  | **24** | Availability of mentoring and supervision for physical health assessment and intervention. | 1 | 1 |
|  | **25** | Prompts and processes for ensuring physical health needs are revisited regularly. | 1 | 1 |
|  | **26** | Normalised use of accessible language for describing physical health priorities, including assessment and intervention. | 0 | 0 |
| Motivation | **1** | Believing that physical healthcare can contribute to mental health recovery. | 46 | 11 |
|  | **2** | Physical healthcare has become habitual and integrated into daily practice. | 38 | 12 |
|  | **3** | Influence of role models or champions in physical healthcare. | 38 | 12 |
|  | **4** | Physical healthcare leadership identity in mental health settings. | 33 | 10 |
|  | **5** | Professional confidence in working effectively with consumers to facilitate behaviour change. | 29 | 10 |
|  | **6** | Valuing and prioritising physical healthcare of consumers. | 27 | 10 |
|  | **7** | Confidence in providing physical healthcare in mental health settings. | 22 | 7 |
|  | **8** | Reinforcement (incentives/consequences) for staff to provide physical healthcare. | 21 | 10 |
|  | **9** | Individual staff commitment to delivering physical healthcare (personal planning, intentions, goals) in their role. | 11 | 5 |
|  | **10** | Managing negative responses, such as stress, anxiety, burn-out. | 11 | 4 |
|  | **11** | Social/professional role identity prioritises physical health. | 9 | 4 |
